# A Transcript and Metabolite Atlas of Blackcurrant Fruit Development Highlights Hormonal Regulation and Reveals the Role of Key Transcription Factors

**DOI:** 10.3389/fpls.2018.01235

**Published:** 2018-08-24

**Authors:** Dorota A. Jarret, Jenny Morris, Danny W. Cullen, Sandra L. Gordon, Susan R. Verrall, Linda Milne, Pete E. Hedley, J. William Allwood, Rex M. Brennan, Robert D. Hancock

**Affiliations:** ^1^James Hutton Limited, Dundee, United Kingdom; ^2^Cell and Molecular Sciences, The James Hutton Institute, Dundee, United Kingdom; ^3^Information and Computational Sciences, The James Hutton Institute, Dundee, United Kingdom; ^4^Environmental and Biochemical Sciences, The James Hutton Institute, Dundee, United Kingdom

**Keywords:** metabolomics, fruit ripening, non-climacteric fruit, organ development, endoreduplication

## Abstract

Blackcurrant fruit collected at six stages of development were assessed for changes in gene expression using custom whole transcriptome microarrays and for variation in metabolite content using a combination of liquid chromatography-mass spectrometry and gas chromatography-mass spectrometry. Principal components analysis demonstrated that fruit development could be clearly defined according to their transcript or metabolite profiles. During early developmental stages, metabolite profiles were dominated by amino acids and tannins, whilst transcript profiles were enriched in functions associated with cell division, anatomical structure morphogenesis and cell wall metabolism. During mid fruit development, fatty acids accumulated and transcript profiles were consistent with seed and embryo development. At the later stages, sugars and anthocyanins accumulated consistent with transcript profiles that were associated with secondary metabolism. Transcript data also indicated active signaling during later stages of fruit development. A targeted analysis of signaling networks revealed a dynamic activation and repression of almost 60 different transcripts encoding transcription factors across the course of fruit development, many of which have been demonstrated as pivotal to controlling such processes in other species. Transcripts associated with cytokinin and gibberellin were highly abundant at early fruit development, whilst those associated with ABA and ethylene tended to be more abundant at later stages. The data presented here provides an insight into fruit development in blackcurrant and provides a foundation for further work in the elucidation of the genetic basis of fruit quality.

## Introduction

Blackcurrant (*Ribes nigrum* L.) is a perennial bush native to northern Europe, Scandinavia, and the Russian federation (Hummer and Dale, 2010). It is widely cultivated across the temperate zones of Europe, Russia and New Zealand (Brennan, 2008) where fruit are primarily used for juice production (Brennan and Graham, 2009). Blackcurrant, along with other related currant producing species and also gooseberries, belongs to the *Grossulariaceae* (Messinger et al., 1999) and hence is only distantly related to other crop plants meaning there are few suitable models to base genetic analyses.

In order to overcome these limitations, significant effort has gone into the development of blackcurrant genetic and genomic resources over the last two decades. Early work focussed on the development of random amplified polymorphic DNA (RAPD), inter-simple-sequence repeat (ISSR) and microsatellite (SSR) markers (Lanham et al., 1995, 2000; Brennan et al., 2002). The first linkage map, based on amplified fragment length polymorphism (AFLP), SSR and single nucleotide polymorphism (SNP) markers, was published in 2008 and a number of loci associated with fruit quality, agronomic (Brennan et al., 2008) and pest resistance traits (Brennan et al., 2009) were defined. As technologies have advanced, greater amounts of cDNA and genomic sequence have become available and these sequences have been used both for the design of microarrays (Hedley et al., 2010) and for the development of linkage maps with greater marker density (Russell et al., 2011, 2014). Most recently, several microRNAs and their putative targets have been identified (Xie et al., 2016). Although a great deal of sequence information has become available over the last decade, a key limitation to exploitation remains the accurate annotation of transcripts and the identity of gene function in a species which remains evolutionarily distant from more intensively studied fruit species.

Ripening of fruit is a dynamic and complex process that involves coordinated regulation of development, physiology and metabolism, leading to the production of a soft and edible fruit with desirable quality attributes (Giovannoni, 2001). During ripening, fruit undergo significant physiological and functional transitions, from an organ evolved to deter frugivores and protect developing seeds and embryos to one that functions to attract herbivores to aid seed dispersal. These changes in function are strictly developmentally controlled through hormonal and other signaling pathways, making the developing fruit an excellent model for the study of plant developmental processes.

A large number of studies have adopted systems approaches to the understanding of fruit development, focussing on changes in metabolite content and transcript abundance to build integrated network models and highlight the key regulators in the coordination of fruit development. Examples of such work include studies undertaken in tomato (Carrari et al., 2006), strawberry (Fait et al., 2008), grape (Fortes et al., 2011), peach (Trainotti et al., 2006; Lombardo et al., 2011), blueberry (Zifkin et al., 2012), pepper (Osorio et al., 2012), mango (Deshpande, 2017), citrus (Wang et al., 2017; Zhang et al., 2017), pear (Liu et al., 2018), kiwifruit (Asiche et al., 2018), watermelon (Gao et al., 2018), and olive (Galla et al., 2009). These studies have identified significant processes associated with fruit development and have highlighted key regulatory networks. Such studies provide evidence for the involvement of specific gene products in fruit development and provide further confidence in gene annotation based solely on homology searches. However, a systematic and global analysis of transcript and metabolite changes during development of the evolutionarily distant *Ribes* fruits has not been previously undertaken.

Several studies have been conducted concerning the nutritional profile of ripe blackcurrant fruit in relation to quality (Hancock et al., 2007; Nwankno et al., 2012; Vagiri et al., 2013; Mikulic-Petkovsek et al., 2015; Mattila et al., 2016; Woznicki et al., 2016; Jung et al., 2017), however, much less is known regarding the dynamics of fruit chemistry during development. Furthermore, little is known regarding the underlying molecular mechanisms and patterns of gene expression associated with phytochemical accumulation in blackcurrant fruit.

The motivation for the present study was to increase knowledge regarding the metabolomic and transcriptional profiles of blackcurrant fruit during development. This provides key information regarding the underlying developmental processes and adds to the growing literature concerning development of other fruit species. We further identify key developmental stages associated with the accumulation of components that influence fruit quality. The data also provides improved annotation of blackcurrant transcripts.

## Materials and Methods

### Fruit Sampling and Phenotyping

Fruit of cultivar Ben Finlay was collected at six stages of development from plants grown at the James Hutton Institute, Dundee, United Kingdom (56°27′N, 3°04′W) under standard industry regimes for fertilizer and pest control in 2012. Fruit development was visually assessed as small green (<6 mm), large green (>6 mm), green–red, red–green, red and ripe (Viola et al., 2000) collected on 4 May, 24 May, 3 June, 10 June, 22 June, and 24 July, respectively. Samples were immediately frozen in liquid nitrogen and subsequently stored at -80°C.

Fruit phenotyping was undertaken within 1 h of harvest on fresh fruit samples. Average fruit mass was estimated as 100 berry weight and average fruit diameter measured transversally across the broadest part of thirteen fruit. Fruit dry matter was estimated in a sample of 100 fruit that were weighed before and after lyophilisation. Fruit firmness was estimated by compression using a QTS-25 Texture Analyzer (Brookfield Engineering, United Kingdom) following the manufacturer’s guidelines. The analyzer was fitted with a 25 Kg load cell to drive an aluminum probe of diameter 40 mm and length 80 mm using a trigger point at 0.049 Newtons [N] and probe speed of 30 mm/min to compress the fruit to a level of 20% deformation. Two parameters were determined. Work done expressed in Joules described the total amount of energy required to compress the fruit where a higher quantity of energy is required to compress a firmer fruit. Peak load (N) described the highest amount of force required to maintain constant movement of the probe and again a firmer fruit would require a greater peak load. Total soluble solids and pH were quantified in juice prepared from the different fruit stages as previously described (Nwankno et al., 2012). Fruit chlorophyll and carotenoids were extracted from lyophilised fruit samples and quantified by spectrophotometry as previously described (Hancock et al., 2014).

### Fruit Extraction and Metabolite Analysis

For the preparation of fruit extracts for the analysis of sugars by HPAEC-PAD, organic acids by HPAEC-CD, and polyphenols and anthocyanins by HPLC-MS or spectrophotometric techniques, frozen fruit were ground to a powder in liquid N_2_ and then extracted in 50% (v/v) methanol containing 1% (v/v) formic acid, 2 μM reserpine and 0.5 mM morin at a ratio of 10 ml g FW^-1^. Sugars, organic acids, total polyphenols and total anthocyanins were analyzed as previously described (Nwankno et al., 2012). Individual polyphenols and anthocyanins were analyzed by LC-MS in negative and positive ion modes using the method described by Gavrilova et al. (2011). Compounds were identified using a combination of their relative retention, UV spectra and parent and daughter ion masses as previously described (Määttä et al., 2003; Gavrilova et al., 2011). Relative quantification of compounds was achieved by normalization to the appropriate positive (reserpine) or negative (morin) ion mode internal standard. For the preparation of fruit extracts for the quantification of ascorbate, frozen fruit was ground to a powder in a mortar and pestle with liquid nitrogen and extracted in 5% (w/v) metaphosphoric acid containing 5 mM tris(2-carboxyethyl)phosphine hydrochloride at a ratio of 19:1 (v/w). Following centrifugation, samples were incubated at 5°C for at least 4 h to allow complete reduction of dehydroascorbate to ascorbate (Walker et al., 2006) prior to quantification by HPLC-DAD at 245 nm against known standard concentrations (Nwankno et al., 2012).

Gas chromatography/mass spectrometry (GC/MS) analysis was performed on extracts from three biological replicates per developmental stage, essentially as described by Foito et al. (2013) with modifications for fruit samples as described by Sweetman et al. (2014). Three samples of fruit from independent biological replicates (different plants) were collected as described above then lyophilized. Dried material (100 ± 5 mg) was weighed into glass tubes and extracted in 3 mL methanol for 30 min at 30°C with agitation (1500 rpm). 0.1 mL each of polar (ribitol 2 mg mL^-1^) and non-polar (non-adecanoic acid methyl ester 0.2 mg mL^-1^) standards were added with 0.75 mL distilled H_2_O, and extraction continued for a further 30 min as described. 6 mL of chloroform were added, and extraction continued for 30 min under increased agitation at 2500 rpm. Phase separation was achieved by the addition of a further 1.5 mL of water and centrifugation at 1000 *g* for 10 min. Following oximation, polar metabolites were converted to trimethylsilyl derivatives, while non-polar metabolites were subjected to methanolysis and trimethylsilylation. Polar metabolites primarily comprised amino acids, carbohydrates and organic acids while non-polar metabolites primarily comprised fatty acids, fatty alcohols and phytosterols. Metabolite profiles for the polar and non-polar fractions were acquired following separation of compounds on a DB5-MSTM column (15 m × 0.25 mm × 0.25 μm; J&W, Folsom, CA, United States) using a Thermo Finnigan (San Jose, CA, United States) DSQII GC/MS system as described (Foito et al., 2013). Data were processed using the XCALIBUR software (Thermo Fisher Scientific, Waltham, MA, United States). Peak areas relative to internal standard (response ratios) were calculated following normalization to 100 mg extracted material. Metabolites were identified by comparison to retention indices and mass spectra generated from authentic standards and stored in an in house library.

### RNA Extraction

RNA was extracted from developing fruit using the method previously described (Walker et al., 2010), with the exception that 2 g of fruit were used for all developmental stages and the ratio of extraction buffer to material was reduced to 6:1 in order to increase RNA yield.

### Microarray Design and Application

A custom Agilent gene expression microarray was designed using 2GS RNA-derived sequence data (array design A-MEXP_2372^1^). Briefly, ∼80 million short (75 bp paired-end) Illumina reads were generated from RNA of blackcurrant developing bud tissue (Russell et al., 2011) and assembled into ∼74,000 contiguous transcripts (contigs) using Trinity assembly software^2^. Orientation of contigs were determined by BLASTx homology searches against known genes/proteins in the Arabidopsis genome (TAIR v10) or the *Vitis vinifera* genome database^3^, enabling defined orientation for ∼19,000 transcripts. In addition, ∼21,000 transcripts with unknown orientation were taken forward for array probe design. Single probes (60 mers) were designed to each transcript (one for each orientation for ambiguous sequences) using default parameters in eArray^4^ and formatted into a 60k array design.

The experimental design and complete datasets are available at ArrayExpress^5^ (accession # E-MTAB-6973). Briefly, a single-channel microarray design was utilized with all RNA samples labeled as cRNA with Cy3 fluorescent dye. A total of 18 microarrays were processed, consisting of 3 biological replicates for each of the 6 fruit developmental stages. RNA labeling and downstream microarray processing were performed as recommended in the One-Color Microarray-Based Gene Expression Analysis protocol (v. 6.5; Agilent) using the Low Input Quick Amp Labeling kit (Agilent). Following microarray scanning using an Agilent G2505B scanner, data were extracted from images using Feature Extraction (v.10.7.3.1) software. Intensity data and QC metrics were extracted using the recommended FE protocol (GE2_107_Sep09). Entire FE datasets for each array were loaded into GeneSpring (v.7.3) software for further analysis. Data were normalized using default single-channel settings: intensity values were set to a minimum of 0.01, data from each array were normalized to the 50th percentile of all measurements on the array and the signal from each probe was subsequently normalized to the median of its value across all arrays. Unreliable data flagged as absent in 2/3 replicate samples by the FE software were discarded. Statistical filtering of data was performed using ANOVA adjusted with Benjamini and Hochberg multiple testing correction.

### Real-Time Quantitative PCR

Reverse transcription of 1000 ng of RNA per sample was performed using the QuantiTect Reverse Transcription kit (Qiagen) using a combination of oligo d(T) and random hexamers as primers according to the manufacturer’s instructions. cDNA was diluted with sterile distilled water to a concentration of 10 ng μL^-1^. Quantitative RT-PCR was performed using the Roche Universal Probe Library as described (Campbell et al., 2010). The reactions were repeated in triplicate with independent cDNAs. Relative expression levels were calculated using the 2^-ΔΔ*C*_t_^ method (Livak and Schmittgen, 2001) normalized to geometric means of three control genes; a TIP41-like family protein (comp5696), peroxin4 (comp5152) and cytochrome P450 family protein (comp899). These genes were selected on the basis of previous literature (Czechowski et al., 2005) and the stability of their expression profiles on the array and were evaluated as appropriate internal controls using the Genorm algorithm (Vandesompele et al., 2002). Average expression was estimated from three biological replicates of cDNA where each reaction was completed in triplicate providing three technical replicates. Oligonucleotide primers and probes used in qRT-PCR experiments are described in **Supplementary Table S1**.

### Statistical Analysis

In order to understand dynamics of and between the metabolome and transcriptome, a series of statistical analyses were performed using both sets of data. MS data was presented as response ratios calculated as areas of peaks normalized to internal standard areas for metabolites acquired. Where compounds were detected using more than one method they were observed to be highly correlated (*r* > 0.9), and therefore all data points were included in subsequent analysis.

All statistical analyses were performed using Genstat v. 14 (VSN international) unless stated otherwise. An ANOVA *P*-value of <0.005 was used for transcript data to allow a focus on the most highly significant transcripts. For GO analysis a *P*-value of <0.05 was used to ensure capture of significantly enriched functional categories in the relatively small gene sets with Arabidopsis homologs returned from the ANOVA analysis. For the metabolite ANOVA a *P*-value < 0.05 was set to take account of the greater variability within the metabolite dataset with respect to the transcript dataset.

#### Analysis of Variance (ANOVA)

For transcript data, statistical filtering of data was performed using ANOVA (ANalysis Of VAriance, *P*-value < 0.005) adjusted with Benjamini and Hochberg multiple testing correction. Transcript analysis was carried out in Genespring (v. 7.3) for transcriptomic data and Genstat for metabolomic data. All transcript data were log transformed as default in Genespring prior to analysis.

#### *K*-Means Clustering

A set of transcripts obtained by ANOVA *P*-value < 0.005 filtering was subjected to *K*-means clustering to identify transcripts with co-expression patterns using GeneSpring (v.7.3) software at parameters of 6 clusters and 100 iterations using Pearson’s Correlation as a similarity measure.

#### Cluster Analysis of Transcript Data

Cluster analysis was performed for several sets of transcriptome data in order to obtain groups of probes linked depending on their expression patterns. Analysis was performed using GeneSpring (v. 7.3) using similarity measure of Pearson’s correlation with clustering algorithm of average linkage.

#### Hierarchical Clustering of Metabolite Data

Analysis of Variance (ANOVA) was performed on each individual metabolite quantified in order to identify those with significant differences in response between the developmental stages. Only significant metabolites with a *P*-value < 0.05 were analyzed further using cluster analysis. The data was expressed on a standard error of difference (SED) scale from the ANOVA output. Since the SEDs express the differences scaled by their variability, this standardization allows metabolites to be compared with respect to relative change both within and across technologies. Partitioning was based on Hierarchical Cluster Analysis (HCA) with complete linkage and Euclidean distance. Clusters were defined by assessing the compounds within the most similar clusters, initial cut off was 96% similarity, however, further manual adjustment was undertaken to group compounds that were clearly clustered based on visual assessment of the dendrogram. This procedure generated clusters which share the same pattern between developmental stages as defined by significant changes rather than absolute values.

#### GO Terms Enrichment

Individual gene sets identified by *K*-means analysis were analyzed for enrichment of Gene Ontology (GO) terms using the Term Enrichment Tool AgriGo 1.2^6^ (Du et al., 2010). This was enabled by utilizing the top BLAST hit of each blackcurrant transcript to the Arabidopsis TAIR9 gene model database. Gene sets were checked for term enrichment against the same database at default settings of Fisher’s statistical method, Yekutieli (FDR under dependency) multi-test adjustment method and *P*-value < 0.05 level of significance.

#### Principal Component Analysis (PCA)

Principal component analysis (PCA) was performed using the 1433 differentially expressed transcripts (ANOVA *P*-value < 0.005) obtained from microarray expression study and all 194 metabolites obtained by several quality quantifying methods. PCA can be carried out using either the sample variance-covariance matrix or the sample correlation matrix: the former focuses on the most abundant metabolites, while the latter gives equal weight to all metabolites. Transcript data was log transformed and analyzed using variance-covariance matrix using Genstat. Metabolite data was checked for normal distribution and analyzed using a sample correlation matrix.

## Results

### Fruit Developmental Profiles

**FIGURE 1:**
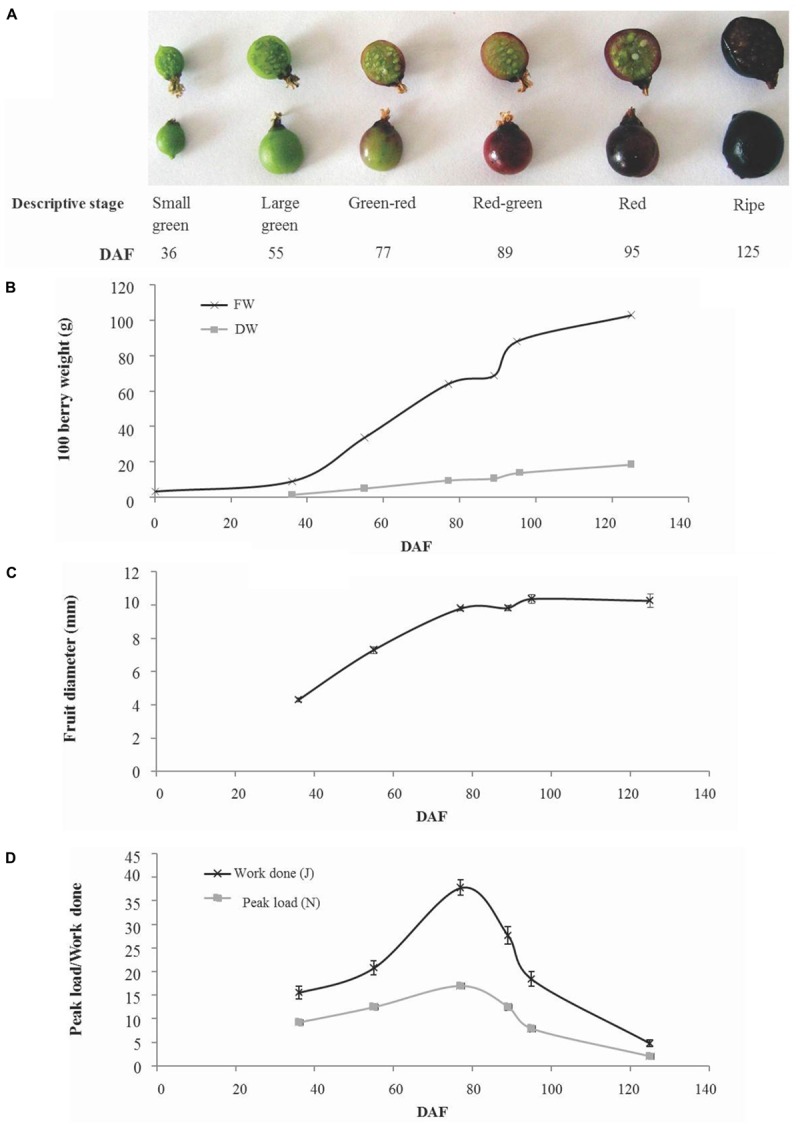
Developmental phenotype of blackcurrant fruit. Representative fruit harvested at numbered days after flowering (DAF) of six descriptive stages are illustrated **(A)**. The fresh and dry weight of flowers and fruit were recorded for 100 fruit **(B)** and the mean fruit diameter (*n* = 13) was measured and is indicated ± SE **(C)**. Fruit firmness **(D)** was estimated as the peak load required or total work done to compress whole fruit by 20% and is indicated as mean ± SE (*n* = 13).

Fruit development took a total of 125 days from open flowers to ripe fruit and occurred over the period March 29– August 1, 2012. Fruit were collected at six developmental stages that cover the major developmental transitions (**Figure 1A**). Fruit fresh weight increased in a linear fashion from the small green (36 days after flowering; DAF) up to the green–red stage (77 DAF). This was followed by a pause in fresh weight gain to the red–green stage (89 days after flowering) then a rapid increase in fresh weight gain to the red fruit stage (95 days after flowering) and a more gradual increase to maturity (125 days after flowering). A similar pattern was observed in dry weight gain although there was a less pronounced cessation and subsequent increase in dry weight gain around the green–red to red stages of fruit development (**Figure 1B**). Dry matter estimated in a sample of 100 fruit varied between 15% (w/w) in green–red fruit and 18% (w/w) in ripe fruit (**Supplementary Figure S1**). Fruit diameter increased almost linearly between small green and green–red stage (77 days after flowering) after which only minor increases in fruit size were observed (**Figure 1C**). Similarly, fruit firmness increased to the green–red stage after which there was a decline until fruit ripened (**Figure 1D**).

**FIGURE 2:**
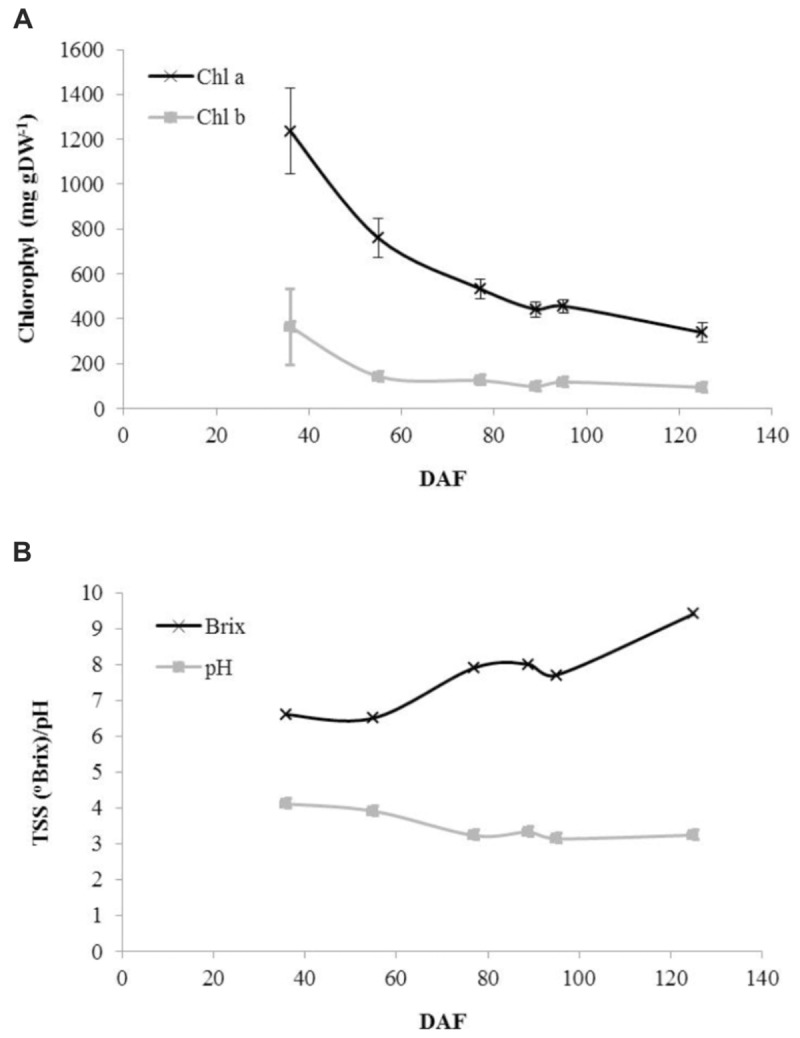
Developmental profiles of fruit pigments and juice characteristics in blackcurrant. Chlorophyll was extracted and quantified from lyophilised fruit as described and is represented as mean ± SE, *n* = 3 **(A)**. Juice Brix and pH values were estimated as described from prepared juice samples **(B)**. DAF, days after flowering.

Consistent with the change from green to red to ripe fruit, a decline in photosynthetic pigment content was observed throughout development although small amounts of both chlorophyll a and chlorophyll b were still present in ripe fruit (**Figure 2A**). Juice soluble solids were constant in green fruit before exhibiting an increase to green–red fruit followed by a further decline and then an increase to a maximum in ripe fruit (**Figure 2B**). Juice pH exhibited a steady decline to the red–green stage after which it was approximately constant around pH 3.25 (**Figure 2B**).

### Fruit at Different Developmental Stages Exhibit Specific Transcript and Metabolite Profiles

**FIGURE 3:**
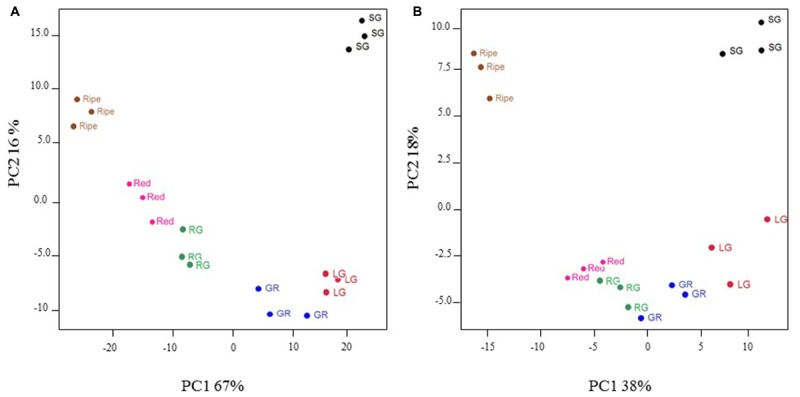
Principal components analysis of significantly differentially expressed transcripts **(A)** and selected metabolites **(B)** in developing blackcurrant fruit. Fruit were collected from three individual plants at the small green (SG), large green (LG), green–red (GR), red–green (RG), red and ripe fruit stages.

Principal components analysis was used to group fruit samples according to the profile of 1432 highly significant (*P* < 0.005) differentially expressed transcripts, or a broad range of primary and secondary metabolites including sugars, organic acids, amino acids, fatty acids, flavonols, and anthocyanins. For transcripts, the first two principal components accounted for 67 and 16%, respectively, of the total variation within the data and clearly separated individual fruit stages as well as clustering replicates of any one fruit stage (**Figure 3A**). Metabolite profiles similarly discriminated fruit developmental stages and the first two principal components accounted for 38 and 18% of the variation, respectively (**Figure 3B**). Green–red, red–green, and red fruit stages clustered more closely on the metabolite PCA plot (**Figure 3B**) than on the transcript plot (**Figure 3A**) indicating that metabolite profiles were more similar than transcript profiles in these fruit developmental stages. Nevertheless, the data presented here validates our fruit sampling protocol and indicates key differences in transcript and metabolite profiles at different stages of development.

### Hierarchical Clustering of Metabolites Reveals Dynamic Changes in Fruit Metabolite Content

**FIGURE 4:**
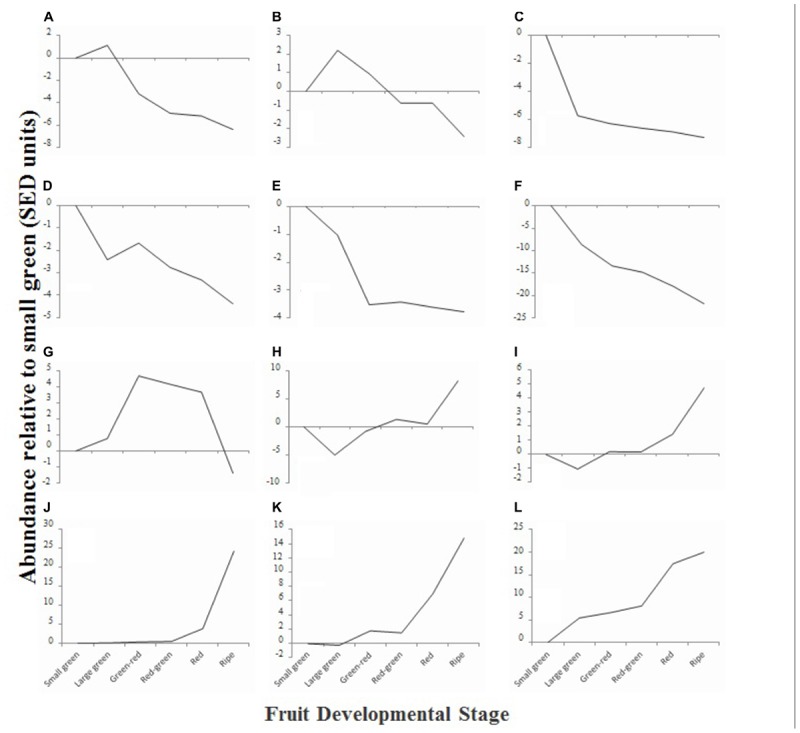
Relative average abundance of metabolite clusters identified by hierarchical cluster analysis at six stages of blackcurrant fruit ripening. Clusters were identified at an initial cut-off of 96% similarity with additional manual adjustment based on visual assessment of similarity dendrogram and are identified in the text as labeled **(A–L)**. Data are represented as the average change of abundance of compounds present in the cluster relative to average abundance in small green fruit. Data were normalized based on standard errors of difference (SED) as described in materials and methods.

To obtain an overview of the phytochemical changes that occur during fruit ripening, we used a combination of analytical techniques to examine the relative or absolute concentrations of a range of compounds that are relevant to fruit quality. Compounds quantified included sugars, organic acids, fatty acids and fatty alcohols, amino acids, flavonoids and anthocyanins. Initial data analysis was achieved by hierarchical clustering. Compounds that exhibited similar changes in concentration profile across development were grouped and 12 clusters (A–L) were identified (**Figure 4**). Six clusters (**Figures 4A–F**) were comprised of compounds that exhibited an overall decline in concentration during fruit maturation, while five clusters (**Figures 4H–L**) were comprised of compounds that exhibited an overall increase in concentration during development. One cluster (**Figure 4G**) was composed of compounds that were most abundant during mid-fruit ripening stages before falling back to levels similar to those in small green fruit in ripe fruit.

Analysis of the composition of the different clusters revealed that in many cases they were composed of compounds that were metabolically related (**Supplementary Table S2**). Clusters A and B, which were composed of compounds that exhibited a concentration peak in large green fruit, were dominated by the presence of amino acids and other amines. These included a range of plastid synthesized amino acids such as methionine, lysine, threonine, isoleucine, and phenylalanine (de la Torre et al., 2014), GS/GOGAT cycle derived amino acids, and non-protein amino acids such as β-alanine and polyamines (**Supplementary Table S2**). Cluster C, comprising compounds that were most abundant at the small green stage, contained a number of tannins and phenolic acids such as chlorogenic and cinnamic acids (**Supplementary Table S2**), while cluster D, that contained compounds that also showed a decline in abundance from small green fruit to ripening, also contained an epi-gallocatechin tannin, the polyphenol myrecetin-hexoside and also included a measure of total polyphenols estimated using a specific enzymatic method (Stevanato et al., 2004). Clusters E and F contained compounds declining in abundance and comprised a large number of unknown compounds identified in the polar extract analyzed by gas chromatography as well as several organic acids, including the TCA cycle intermediates succinate and malate, as well as threonate and quinate (**Supplementary Table S2**).

Compounds in cluster G exhibited an increased concentration during the mid-fruit ripening stages before decreasing in ripe fruit. This cluster was dominated by fatty acids ranging in carbon number from 14 to 18 and included the major seed oil fatty acids palmitate, stearate, linoleate, and linolenate (**Supplementary Table S2**) (Ruiz del Castillo et al., 2002).

Cluster H comprised the major monosaccharides glucose and fructose, as well as the minor sugars galactose and mannose (**Supplementary Table S2**). The concentration of these compounds declined between small and large green fruit before rising to a plateau in green–red, red–green and red fruit and then increasing steeply in ripe fruit. Compounds in cluster I exhibited a similar pattern, however, the concentration of compounds in this cluster were already elevated in red fruit. This cluster comprised a number of long chain fatty acids (C20 – C28) that are associated with cuticular waxes and found at high concentrations in deseeded blackcurrant pomace relative to seed or oil (Dobson et al., 2012) and a number of the flavonols and anthocyanins that are found at relatively low abundance in ripe fruit, such as a kaempferol-hexoside, cyanidin-glucoside, and peonidin-(coumaroyl)glucoside (**Supplementary Table S2**). Clusters J, K, and L contained compounds that exhibited significant increases in concentration toward the latter stages of ripening. These contained the major anthocyanins delphinidin- and cyanidin-rutinoside as well as other less abundant anthocyanins [delphinidin-glucoside, petunidin-(coumaroyl)glucoside] and the flavanol kaempferol-hexoside-deoxyhexoside. Furthermore, a spectrophotometric measure of total anthocyanins localized to cluster K (**Supplementary Table S2**).

### *K*-Means Clustering Reveals Divergent Patterns of Gene Expression During Development

**FIGURE 5:**
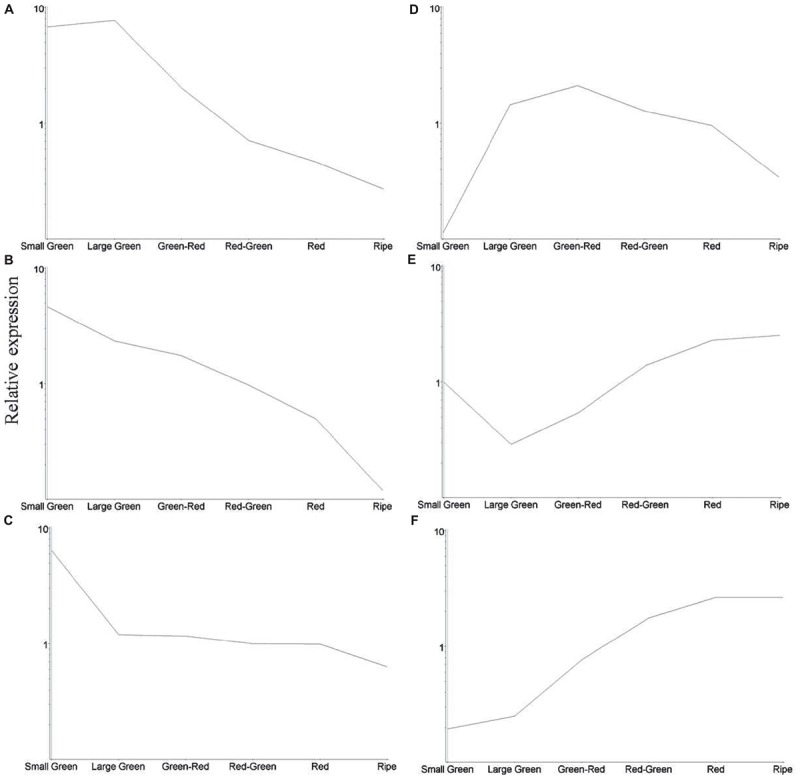
Relative average abundance of transcript clusters identified by *K*-means cluster analysis at six stages of blackcurrant fruit development. Individual clusters are labeled **(A–F)** and described in the text. Relative changes in transcript clusters are represented on a logarithmic scale.

To obtain an overview of significant changes in gene expression during fruit development we identified 1433 differentially expressed transcripts as estimated by analysis of variance, *P* < 0.005 (**Supplementary Table S3**). Transcripts were clustered using the *K*-means algorithm producing six clusters exhibiting similar expression profiles (**Figure 5**). Clusters A, B, and C containing 241, 256, and 273 transcripts, respectively, exhibited a decline in transcript abundance over development. Transcripts in cluster D (141 transcripts) were present at low abundance in small green and ripe fruit, but were more abundant during mid-fruit development. Cluster E (209 transcripts) comprised transcripts that were least abundant at the large green and green–red stages, while transcripts in cluster F (312 transcripts) exhibited an increasing trend in abundance throughout the developmental period.

As a first interrogation of transcript data, GO term enrichment analysis was conducted on annotated transcripts present in the different clusters (**Supplementary Table S4**). Cluster A contained 135 transcripts that had high confidence Arabidopsis homologs. Consistent with active cell division at the early stages of fruit development, transcripts associated with the cell cycle (GO:0007049) were the most significantly enriched in this cluster, with a little over 10% of the transcripts associated with this function compared with less than 1% of genes in the Arabidopsis genome being associated with the same function (**Supplementary Figure S2**). These included transcripts (comp11660, comp3973, comp4449, and comp5241) with homology to a range of cyclins (at1g16330, at1g20610, at5g06150, and at5g11300) and cyclin dependent protein kinases (comp386/at1g20930, comp2647/at2g38620). Consistent with active cell division, GO terms associated with DNA replication (GO:0006260) were also highly significantly overrepresented within this cluster and included transcripts with homology to Arabidopsis transcripts encoding MINICHROMOSOME MAINTENANCE 10 (comp12886/at2g20980) required for the initiation of DNA replication and TELOMERASE REVERSE TRANSCRIPTASE (comp20703/at5g16850).

Of the 256 transcripts associated with cluster B, 153 had significant Arabidopsis homologs. The most significantly enriched GO category in this cluster was response to light (GO:0009416, **Supplementary Table S4**), where approximately 7% of significant annotated transcripts in this cluster were associated, compared with only 1.5% of genes in the Arabidopsis genome (**Supplementary Figure S2**). These included a number of transcripts (comp446, comp2831, comp11926, and comp47425) with homology to Arabidopsis genes involved in red light signal transduction or response (at1g75780, at4g31500, at4g03400, and at4g32289). Several of these genes are also known to be involved in auxin signaling and response (Cluis et al., 2004; Sun et al., 2013; Maharjan et al., 2014) and the gene ontology term response to auxin stimulus (GO:0009733) was also significantly enriched within genes that were expressed within cluster B (**Supplementary Table S4**). Other significant GO terms included cellular developmental processes (GO:0048869) and anatomical structure morphogenesis (GO:0009653). Included in the former were genes homologous to those encoding expansins (comp9500/at4g38400 and comp1051/at4g28250), as well as those required for pollen (comp6131/at2g19070) or embryo development (comp339/at5g23940). The latter included a MADS box transcription factor (comp7052) with homology to Arabidopsis AGL7 (at1g69120) with roles in floral meristem identity and the regulation of cytokinin levels (Han et al., 2014).

Cluster C transcripts exhibited a sharp decline in expression between the small and large green stages of fruit development, followed by a less abrupt decline in abundance throughout the remainder of fruit development. Of the genes exhibiting this pattern of expression 154 had Arabidopsis homologs and the most significantly enriched GO term was cell wall metabolism (GO:0042545, **Supplementary Table S4**), with more than 4.5% of the genes within this cluster associated, compared with less than 0.5% of genes within the Arabidopsis genome (**Supplementary Figure S2**). These included genes encoding proteins with functions in cell wall loosening, such as expansins (comp13986/at2g39700, comp2421/at3g29030), pectate lyases (comp31286/at2g36710, comp12064/at5g19730) and a xyloglucan-xyloglucosyl transferase (comp55230/at1g10550). Another GO term that was significantly enriched was associated with enzyme linked receptor protein signaling pathway (GO:0007167) that was 10-fold more represented in cluster C transcripts than in the Arabidopsis genome (**Supplementary Figure S2**). A role for various hormones in early fruit development was also implied by the finding that transcripts associated with response to hormone stimulus (GO:0009725) were significantly over-represented within cluster C (**Supplementary Table S4** and **Supplementary Figure S2**). These included transcripts with roles in signaling or response to multiple hormones. Of particular interest was comp20486 with homology to Arabidopsis RGA-like 1 a DELLA protein known to interact with gibberellin receptors during fruit set (Gallego-Giraldo et al., 2014) and comp14790 homologous to the TEOSINTE BRANCHED1-CYCLOIDEA-PCF 15 that modulates cytokinin and auxin responses during gynoecium development in Arabidopsis (Lucero et al., 2015).

Transcripts in cluster D exhibited greatest abundance during mid fruit development and, of the 141 transcripts associated with the cluster, 79 had Arabidopsis homologs. GO term enrichment was highly significant for a number of terms associated with development (**Supplementary Table S4**). For example, 20% of the annotated transcripts were associated with anatomical structure development (GO:0048856; **Supplementary Figure S2**). A number of these transcripts were specifically associated with seed development and lipid accumulation and included a transcript (comp17985) with homology to oleosin 2 (at5g40420), a major protein found in seed oil bodies (Miquel et al., 2014), a transcript (comp19411) homologous to FUS3 (at3g26790) a B3 domain transcription factor involved in the regulation of seed development (Wang and Perry, 2013), and a transcript (comp42616) homologous to ABNORMAL LEAF-SHAPE1, a subtilisin-like serine protease required for normal embryonic cuticle formation (Xing et al., 2013). A number of the remaining transcripts were homologous to Arabidopsis genes encoding proteins involved in localized auxin synthesis and distribution these included comp29042, a transcript with homology to SEUSS (at1g43850) a transcriptional co-regulator important for the correct pattern of auxin distribution in plant embryos (Lee et al., 2014), and comp17983 homologous to Arabidopsis AUX1, an auxin transporter that plays a role in female gametophyte development (Panoli et al., 2015). Indeed, one of the significantly overrepresented GO terms was response to hormone stimulus (GO:0009725) and, as well as transcripts associated with auxin synthesis and metabolism, several transcripts associated with ABA responses were present.

The most significantly overrepresented GO terms in Cluster E were associated with responses to stimuli, including both external stimuli (GO:0009605) such as wounding (GO:0009611) and endogenous stimuli (GO:0009719), including auxin (GO:0009733) and other hormones (GO:0009725, **Supplementary Table S4**). In addition, cluster E was enriched in transcripts suggesting active metabolism, transport and compartmentation within the fruit. For example, transcripts associated with the biosynthesis of aromatic compounds (GO:0019438) and amino acid derivatives (GO:0042398) were significantly over represented. Transcripts associated with transport (GO:006810), specifically ATPase coupled transport (GO:0042686), were also significantly over represented.

Cluster F contained 170 transcripts that had homology to Arabidopsis transcripts. The most significantly overrepresented GO terms were associated with post-translational protein modification (GO:0043687) and specifically protein phosphorylation (GO:0006468, **Supplementary Table S4**). Transcripts encoded a range of kinase classes, including leucine-rich repeat, as well as cysteine rich receptor-like kinases, calcium dependent kinases and a number of G-type lectin receptor-like kinases. One of the Ca-dependent protein kinases identified (comp27921) exhibited homology to CALCIUM DEPENDENT PROTEIN KINASE 1 (at5g04870) that is known to phosphorylate phenylalanine ammonia lyase, a key rate limiting enzyme of phenylpropanoid biosynthesis (Cheng et al., 2001) and has been shown to stimulate NADPH oxidase activity, suggesting a potential role for oxidative signaling in fruit maturation (Xing et al., 2001).

Cluster F transcripts were additionally enriched in those associated with secondary metabolism (GO:0019748) and included 5 transcripts specifically associated with phenylpropanoid metabolism (GO:0009698). Interestingly, this group contained several transcripts (comp11039, comp26578, and comp236) with homology to Arabidopsis glutathione *S*-transferases (at3g03190, at1g78320, and at1g02920) that are becoming increasingly recognized as essential for transport of flavonoids into the vacuole (Zhao, 2015). The remaining transcripts clustering within this GO category were primarily associated with terpenoid metabolism.

## Regulatory Networks

### Transcription Factors

**FIGURE 6:**
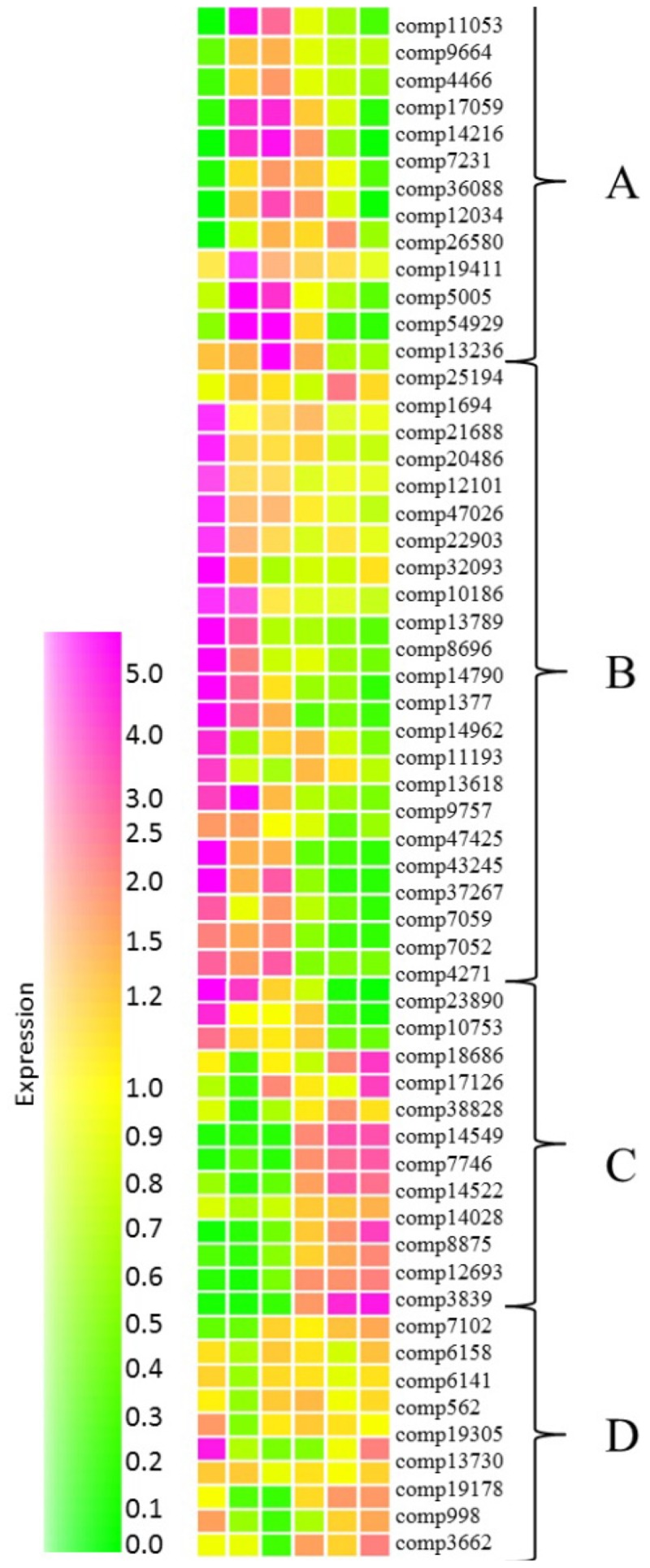
Relative abundance of transcripts encoding putative transcription factors at six stages of blackcurrant fruit development. Transcript levels are expressed relative to the median level of the transcript across all 18 arrays according to the scale indicated. Clusters of transcripts most abundant at mid-fruit development **(A)**, early fruit development **(B)**, late fruit development **(C)**, and both early and late fruit development **(D)** are indicated. Transcripts are identified by their contig reference as indicated to the right of each row.

In order to gain an insight of networks controlling fruit ripening, expression of transcription factor homologs from grape and Arabidopsis databases were investigated. Using this method we identified a total of 57 transcription factor homologs (**Supplementary Table S5**) which were subjected to cluster analysis performed in Genespring. Homologs grouped into four distinctive groups with expression peaking during mid-fruit ripening (**Figure 6**, cluster A), during early fruit development (cluster B), at the later stages of development (cluster C) or at both early and late stages of development with reduced transcript abundance during the middle developmental stages (cluster D).

Many of the transcription factors expressed during the early stages of fruit development (**Figure 6**, cluster B) were homologous to Arabidopsis transcripts encoding transcription factors controlling developmental functions such as mitosis, cell proliferation and differentiation, fruit set and development, and seed development. Interestingly, several of these transcription factors were associated with the process of endoreduplication, where chromosomal division and mitosis are uncoupled resulting in an increase in cell ploidy. Increased ploidy is commonly correlated with increased cell size (Bourdon et al., 2010) and it has been reported that fruit growth in *Ribes* species is driven solely by increased cell volume as cell division ceases following anthesis (Coombe, 1976). Two of the early expressed transcription factors (comp14790 and comp1377) exhibited homology with Arabidopsis TEOSINTE BRANCHED, CYCLOIDEA AND PROFLIFERATING CELL FACTOR (TCP) transcription factors (at3g47620 and at4g18390, respectively). TCP14 (at3g47620) is involved in the control of endoreduplication via the direct regulation of cell cycle genes (Peng et al., 2015), while TCP2 (at4g18390) has been shown to control the expression of NGATHA transcription factors, required for lateral organ growth (Ballester et al., 2015). Similarly, comp14962 with homology to the Arabidopsis E2F transcription factor DEL1 (at3g48160) is known to affect the developmental onset of endoreduplication by directly controlling the expression of cell cycle associated transcripts (Lammens et al., 2008). Similarly, two transcription factors (comp7052 and comp4271) with homology to the MADS box transcription factors AGL7 (at1g60910) and AGL8 (at5g60910) were expressed during early fruit development. AGL7 has roles in flower development in Arabidopsis (Irish and Sussex, 1990) while AGL8, otherwise known as FRUITFULL, mediates cell differentiation during early fruit development (Gu et al., 1998).

A second group of transcription factors that were abundant during early stages of fruit development were homologous to transcription factors with functions in cuticle development. The ethylene response factors SHINE1 (at1g15360), SHINE2 (at5g11190), and the MYB16 transcription factor (at5g15310) work in a coordinated fashion to induce genes required for cuticle synthesis (Aharoni et al., 2004; Oshima et al., 2013) and homologs (comp8696, comp13789, and comp10186) of all three were expressed during the early stages of blackcurrant fruit development. Furthermore, two HD-ZIP transcription factors comp43245 homologous to ANL2 (at4g00730) and comp37267 homologous to HDG1 (at3g61150) were highly expressed in early developing fruit. Tomato plants with a mutation in the homolog of ANL2 have significant defects in the fruit cuticle (Nadakuduti et al., 2012), while HDG1 has been identified as a positive regulator of cuticle development in Arabidopsis and rice (Wu et al., 2011).

Group A comprised 13 transcription factor homologs that were most abundant during mid fruit ripening. Many of these transcription factors encoded proteins homologous to those with functions in seed and embryo development. These included comp5005, a zinc finger homeodomain transcription factor with homology to MATERNAL EMBRYO ARREST 68 (at4g24660). Arabidopsis lines containing a mutation in MEE68 exhibit an arrest of embryo development after a few cell divisions (Pagnussat et al., 2005). Similarly comp9664, a WRKY transcription factor with homology to at5g56270 (WRKY2) was highly expressed during mid fruit development. In Arabidopsis WRKY2 drives the cell specific expression of *WOX8* and *WOX9* homeobox genes that are required for early embryo patterning and *wrky2* mutants exhibit distorted embryo development (Ueda et al., 2011). Several transcription factors that are considered to be global regulators of embryo and seed development were also significantly overexpressed during the middle stages of fruit development. In Arabidopsis the *LEAFY COTYLEDON* group transcription factors LEC1, LEC2, and FUS3 are essential for embryo development (Wang and Perry, 2013). A zinc finger transcription factor (comp12034) that was highly abundant during mid-fruit development was homologous to the Arabidopsis ZF4 (at1g03790) that acts as a negative regulator of seed germination (Bogamuwa and Jang, 2013). Another transcription factor expressed during mid fruit development (comp54929) has homology to at3g27010, a TCP transcription factor gene that participates in the regulation of cell expansion targeting genes involved in cell wall biogenesis and modification (Herve et al., 2009).

Transcription factors in group C comprising a total of 12 transcripts displayed an increasing abundance toward the later stages of fruit development (**Figure 6**). A small number of transcripts with homology to transcription factors involved in developmental control were present in this group (comp23890, comp18686, and comp3839) although their Arabidopsis homologs (at5g39660 encoding CYCLING DOF FACTOR 2, at1g68800 encoding TCP12 and at1g43850 encoding SEUSS) have not been reported to be directly involved in fruit development (Aguilar-Martínez et al., 2007; Fornara et al., 2009; Wynn et al., 2014). Of the remaining transcription factors exhibiting increased abundance at the later stages of fruit development the majority were homologous to Arabidopsis genes responsible for coordinating response to stress. The expression of these transcripts were consistent with hypoxia in enlarging fruit. Comp12693 is homologous to Arabidopsis WRKY75 (at5g13080) while comp10753 shows homology to Arabidopsis HYPOXIA RESPONSE ATTENUATOR1 (at3g10040). WRKY75 increases in both abundance and ribosomal occupancy following exposure of Arabidopsis to hypoxia stress (Branco-Price et al., 2005), while HRA1 fine tunes the hypoxia response (Giuntoli et al., 2014).

Group D transcription factors exhibited a pattern of expression where they were highly abundant during early and late fruit development but less abundant during the mid-phases of development, particularly at the large green stage (**Figure 6**). The eight transcription factors identified in this group exhibited diverse functions, however, several transcription factors in group D had roles in cell fate and development. These included comp19178 and comp13730 that were homologous to Arabidopsis WOX4 (at1g46480) and WRKY23 (at2g47260), respectively. WOX4 has a function in the definition of the vascular stem cell niche (Zhou et al., 2015) while the controlled expression of WRKY23 is required for appropriate stem cell speciation and also regulates the biosynthesis of quercetin (Grunewald et al., 2013). The abundance of comp19305 was high both at the small green and the red-ripe stages of development (**Figure 6**). This transcription factor exhibits homology to an Arabidopsis tandem zinc finger transcription factor (at1g66810) that has roles in cell elongation via promotion of the expression of genes required for secondary cell wall biosynthesis and modification (Kim et al., 2014) suggesting a role both in cell wall biosynthesis during the early stages of fruit expansion and modification during the fruit softening stage.

### Hormones

The role of a range of hormones in different aspects of fruit development is now well established and a consensus on the key hormones involved across a range of dry and fleshy fruit has now been achieved. Fruit set has been attributed to the actions of auxin, gibberellin and cytokinin; fruit growth and cell expansion is regulated by auxin, gibberellin and ABA; while ripening appears to be primarily under the control of ABA and ethylene (McAtee et al., 2013).

In the present study, we interrogated the dataset of differentially expressed transcripts obtained from applying an ANOVA cut off value of *p* ≤ 0.05. From this dataset of 4714 transcripts we then identified the subset that were homologous to Arabidopsis transcripts associated with hormone synthesis, metabolism or signaling to examine their patterns of expression during fruit development.

**FIGURE 7:**
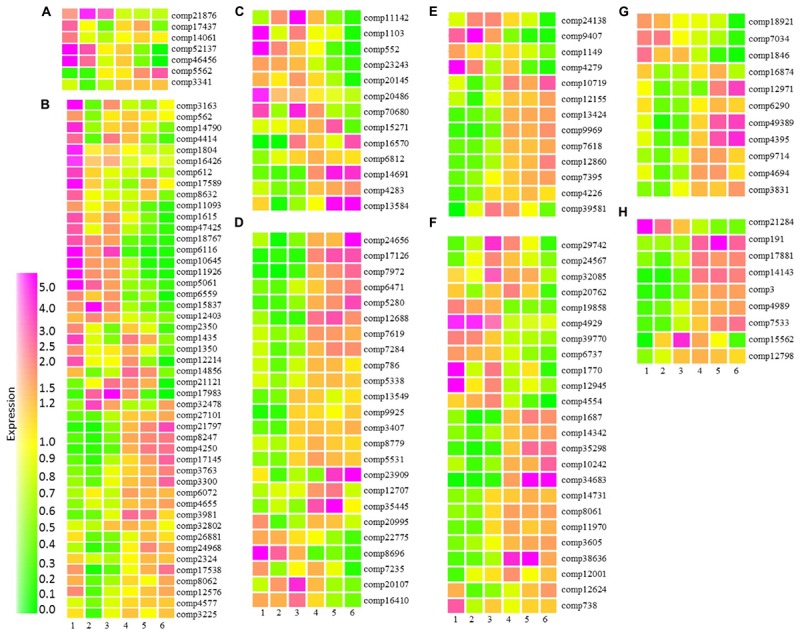
Relative abundance of transcripts encoding proteins associated with hormone metabolism and signaling at six stages of blackcurrant fruit development. Transcript levels are expressed relative to the median level of the transcript across all 18 arrays according to the scale indicated. Transcripts shown are putatively associated with cytokinin **(A)**, auxin **(B)**, gibberellin **(C)**, ethylene **(D)**, abscisic acid **(E)**, brassinosteroid **(F)**, jasmonate **(G)**, and salicylate **(H)** metabolism or signaling. Transcripts are identified by their contig reference as indicated to the right of each row.

Cytokinin is widely accepted as being a key hormone regulating fruit set and is also involved in the cell division stages of fruit development (McAtee et al., 2013; Kumar et al., 2014). In tomato cytokinin ribosides were high at anthesis, while high levels of *trans*-zeatin were associated with early cell division (Matsuo et al., 2012). We identified seven transcripts with homology to Arabidopsis transcripts associated with cytokinin metabolism or response. Five of these transcripts were highly expressed during early fruit developmental stages (**Figure 7A**). These included three transcripts with homology to cytokinin oxidases involved in the degradation of cytokinins and two transcripts associated with cytokinin response (**Supplementary Table S6A**). The observation that cytokinin oxidases were already very highly expressed at the small green stage of fruit development is consistent with a role for cytokinin in fruit set. Furthermore, the data supports the suggestion that in blackcurrant increases in fruit size are driven by cell expansion rather than cell division where cytokinin degradation shortly after fruit set may inhibit cell division. Transcripts with homology to the Arabidopsis cytokinin sensors histidine kinase 2 (HK2) and HK3 were more highly expressed in the later stages of fruit development (**Figure 7A** and **Supplementary Table S6A**). In Arabidopsis roots both HK2 and HK3 transcripts are increased by a range of abiotic stresses (Singh et al., 2015) and the observed increase in transcript abundance during the latter stages of fruit development may represent increased abiotic stress as fruit start to mature and enter senescence.

Auxin and gibberellin are also known to play an interdependent role in fruit set and early development, where auxin is able to induce gibberellin biosynthesis (McAtee et al., 2013). We identified 46 transcripts associated with auxin metabolism and signaling that were significantly differentially expressed throughout fruit development in blackcurrant (**Figure 7B** and **Supplementary Table S6B**). Thirty transcripts were highly expressed at the small green stage of development, these included a number of auxin-induced and small auxin-upregulated RNA (SAUR) and SAUR-like transcripts, as well as auxin efflux carriers with homology to transcripts encoding Arabidopsis PIN-FORMED (PIN) proteins, such as PIN1, PIN3, PIN6, and PIN 7. Early abundant transcripts also exhibited homology to a number of Arabidopsis transcripts encoding auxin biosynthetic or metabolic proteins. Approximately 20 transcripts were highly expressed at the later stages of fruit development during fruit maturation and ripening (**Figure 7B**). A small number of these transcripts had homology to Arabidopsis transcripts encoding SAUR and other auxin responsive proteins (**Supplementary Table S6B**). There were also a number of transcripts with homology to Arabidopsis transcription factors, such as AUXIN RESPONSE FACTOR(ARF)5 with functions in embryo development and ARF1 which has been implicated in controlling aspects of maturation and senescence.

Thirteen transcripts were identified as being involved in gibberellin metabolism or signal transduction. Like the transcripts associated with auxin, there was a roughly even split between transcripts that were expressed in the early stages of fruit development and those expressed later in development (**Figure 7C**). A transcript (comp7068) with homology to an Arabidopsis transcript encoding GA REQUIRING1 (GA1, at4g02780) required for one of the plastid localized early steps of GA biosynthesis was highly abundant at the earliest stage of fruit development and then exhibited a second peak of abundance during mid-fruit ripening as seeds were developing and maturing (**Figure 7C** and **Supplementary Table S6C**). Two further biosynthetic transcripts encoding GA20 oxidases showed similar but distinct patterns of expression. GA20 oxidases are present in the cytosol and catalyze several steps in the biosynthesis of the immediate precursors of the active forms of gibberellin, GA1 and GA4 (Hedden and Thomas, 2012). Two GA2 oxidases required for the catabolism of active GAs were also present within the significantly differentially expressed group of transcripts. Comp23243 homologous to GA2OX6 (at1g02400) was highly abundant in green and green–red fruit, while comp15271 homologous to GA2OX2 (at1g30040) in green–red, red–green and particularly red fruit.

Some 24 blackcurrant transcripts had homologs to Arabidopsis transcripts associated with ethylene (**Figure 7D** and **Supplementary Table S6D**). The role of ethylene in ripening has been studied extensively. In climacteric fruit a burst of ethylene biosynthesis is characteristic of the ripening process (Giovannoni, 2004), but non-climacteric fruits show no increase in respiration and ethylene production during ripening. However, low levels of ethylene may be involved in the development and aspects of ripening (Trainotti et al., 2005; Mu et al., 2016). The majority of blackcurrant transcripts were highly expressed in late stages of fruit development (**Figure 7D**). Those included a range of ethylene receptors ETR2 (comp6471/at3g23150), EIN4 (comp7284/at3g04580), and ERS1 (comp5338/at2g40940), having functions of ethylene sensing (Hall et al., 2000). This is in line with grape transcript abundance of several ethylene receptors and indicates some similarities in the perception and the integration of the ethylene signaling with what was already observed in climacteric fruits (Chervin and Deluc, 2010). Also highly expressed in green–red, red–green, red and ripe stages and involved in regulation of flavonol biosynthesis (Lewis et al., 2011) was EIN2 (comp3407/at5g03280). In addition to receptors and signaling proteins, two transcripts encoding ACC synthase homologs (comp12688, comp8779) required for the biosynthesis of the immediate precursor of ethylene, were highly upregulated in the final stages of fruit development. In contrast to ACC synthases, ACC OXIDASE 1 (ACO1) required for the conversion of ACC to ethylene was highly expressed in the first stage in addition to late stages of fruit development, which in early stages is true for grape (Deluc et al., 2007) and in late stages in watermelon (Wechter et al., 2008).

Alongside ethylene, ABA is a key regulator of fruit ripening in both climacteric and non-climacteric fruits with roles in the control of sugar accumulation, flavonoid biosynthesis and cell wall softening (Leng et al., 2014). Increases in ABA content have been observed in a large number of fruit prior to the onset of ripening and the accumulation of ethylene. We observed significant differences in the abundance of 13 transcripts that exhibited homology to Arabidopsis transcripts associated with ABA synthesis and signaling during the development of blackcurrant fruit. Four of these transcripts were expressed during the earlier phases of fruit development (**Figure 7E** and **Supplementary Table S6E**). These included comp9407, exhibiting homology to an Arabidopsis transcript (at4g19170) encoding CAROTENOID CLEAVAGE DIOXYGENASE4 (CCD4), a carotenoid cleavage dioxygenase with close homology to the 9-*cis*-epoxycarotenoid cleavage dioxygenases that catalyze a key step in ABA synthesis (Auldridge et al., 2006). In Arabidopsis, CCD4 is highly expressed late in seed development (Gonzalez-Jorge et al., 2013) consistent with high levels of expression during the small-green to green–red stages of fruit development in blackcurrant (**Figure 7E**). A second transcript, comp4279, encoding a putative biosynthetic protein had homology to an Arabidopsis transcript encoding ABA DEFICIENT2 (ABA2, at1g52340) encoding an ABA aldehyde oxidase required for the final step of ABA biosynthesis (Leng et al., 2014). This transcript was highly abundant at the small and large green stages of fruit development but also at the red–green stage consistent with increased ABA content in fruit at the onset of ripening. A second transcript (comp12155) with homology to at2g27150 encoding ABA ALDEHYDE OXIDASE3 (AAO3) was highly abundant from the red–green through the ripe stages of fruit development (**Figure 7E**).

Several transcripts encoding proteins required for ABA signal transduction were found to be significantly differentially abundant during fruit development. Two transcripts encoding putative PP2Cs, negative regulators of ABA signaling (Cutler et al., 2010), were significantly differentially expressed across fruit development. Comp39581, with homology to at1g07430 encoding HIGHLY ABA-INDUCED PP2C GENE2 (HAI2), was highly expressed at the green–red and red–green stages, while comp10719 homologous to at4g26080 encoding ABA-INSENTIVE1 was highly expressed from the red–green through to the ripe stages of development co-incident with the expression of an AAO3 homolog (**Figure 7E**).

A total of 27 transcripts had homology to proteins associated with brassinosteroid biosynthesis in Arabidopsis (**Figure 7F** and **Supplementary Table S6F**). Brassinosteroids play an important role in aspects of plant physiological development including cell elongation, division, vascular differentiation and flowering (Clouse, 2011). Several studies have shown brassinosteroid involvement in fleshy fruit development and ripening, such as in tomato (Lisso et al., 2006) and grape (Symons et al., 2006). In the present dataset, Arabidopsis homologs included those encoding proteins required for early biosynthetic steps, DWF4, CYP90D1, ROT3. Those homologs were expressed in early stages of fruit development. The proteins from the final steps of brassinosteroid biosynthesis, including BRASSINOSTEROID-6-OXIDASE 2 (BR6OX2), were expressed highly in the first, fifth, and sixth stage of fruit development (**Supplementary Table S6F**). BR6OX2 might be particularly important as it has been demonstrated that VvBR6OX1 had a significant role in promotion of ripening in grape (Symons et al., 2006). Set G (**Figure 7** and **Supplementary Table S6G**) included 11 blackcurrant probes homologous to Arabidopsis proteins associated with jasmonate biosynthesis and signaling. Jasmonate regulates a wide range of processes including plant development such as flowering, tuberization and fruit ripening, and response to environmental factors such as pest and pathogen attack, drought and wounding (Farmer and Ryan, 1990). Three main patterns of expression were identified in this set, transcripts expressed in early stages of fruit development included homologs of JAZ6 (at1g72450), JAZ10 (at5g13220), and LOX2 (at3g45140), involved in jasmonate signal transduction and biosynthesis, respectively. Two further biosynthetic lipoxygenases (LOX1 and LOX5) and another signal transducing JASMONATE-ZIM-DOMAIN PROTEIN (JAZ1) were highly represented in both the first and later (3–6) stages of fruit development.

Salicylic acid (SA) (**Figure 7H** and **Supplementary Table S6H**), has direct involvement in plant growth, flower induction, and uptake of ions, and it has a plant immune signaling function under pathogen challenge. However, it also affects ethylene biosynthesis and enhancement of plant photosynthetic pigments such as chlorophyll and carotenoids (Hayat et al., 2007). Nine blackcurrant transcripts had homology to Arabidopsis SA associated proteins, including immune signal receptor NPR3 (at5g45110) (Fu et al., 2012), three TGA transcription factors (TGA2, TGA4, and TGA9) and PR1 induced in response to a variety of pathogens. All of these homologs were highly expressed in late stages of fruit development (stages 3–6).

### Microarray Validation

In order to validate the expression profiles generated using the microarray platform for *Ribes*, qRT-PCR was performed on six selected genes using gene-specific primers (**Supplementary Table S1**). Each gene represented a divergent expression pattern of one *K*-means cluster (**Figure 5**) which are presented in the same order (**Supplementary Figure S3**). Transcript abundance patterns were calculated along the course of berry development. The geNorm software indicated that UBC and TIP41-like reference genes were the most suitable for accurate normalization for blackcurrant. Comparison of gene expression profiles between microarray data and real-time qRT-PCR presented in **Supplementary Figure S3** indicated clear correspondence of the two independent expression technologies.

## Discussion

**FIGURE 8:**
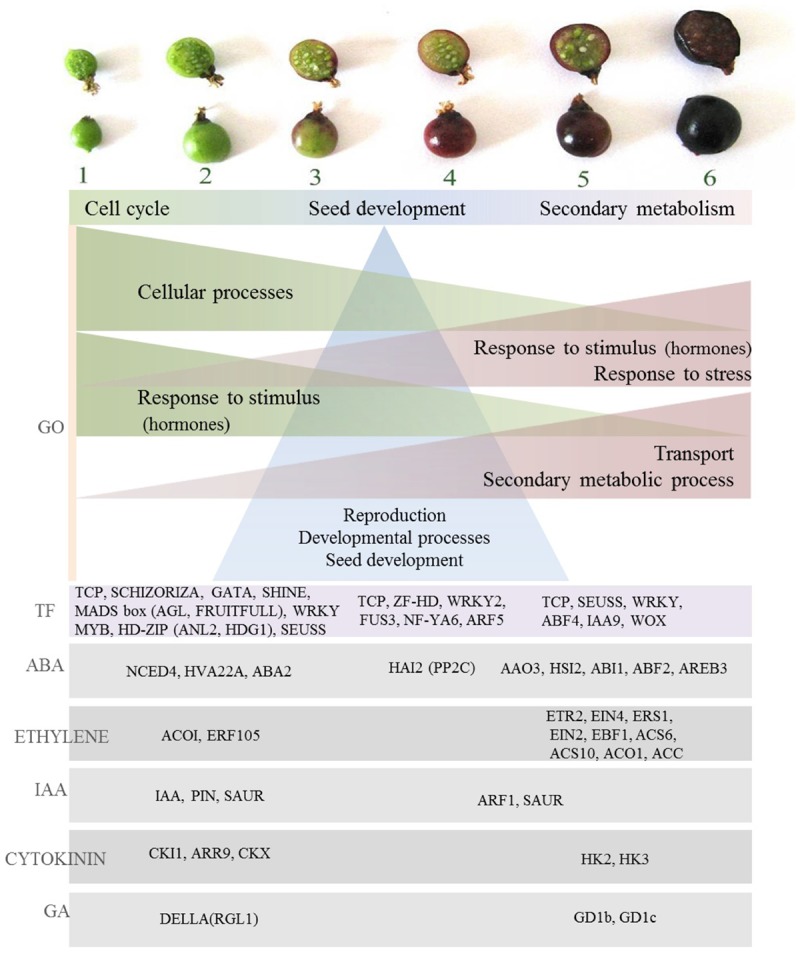
Overview of gene expression associated with fruit development in blackcurrant. Gene ontology (GO) term enrichment highlights specific processes associated with different stages of fruit development. An analysis of transcript abundance at different stages of development highlights the role of specific transcription factors and various transcripts associated with different hormonal signals.

A comprehensive understanding of the regulatory processes of fruit ripening is a vital step in the development of improved methods for controlling quality associated traits and has been elucidated in many fruits such as tomato, apple, pear, grape, and strawberry (Ampopho et al., 2013). Several approaches of gene identification and expression have made a major contribution to the understanding of the molecular basis of fruit quality and ripening control (Grierson, 2013). Hormonal, transcriptional and regulatory networks have been studied and contributed to the identification of important regulatory networks and pivotal genes influencing the ripening process such as MADS-box genes at the Rin (ripening inhibitor) locus necessary for tomato fruit ripening (Vrebalov et al., 2002). Phenotypic analysis of developing blackcurrant fruit has provided an overview of the magnitude of physiological and metabolomic changes during fruit development, in particular, identification of major transitions in fruit growth and metabolite abundancy, accumulation patterns and insights into metabolic regulation. In summary, compounds produced during the first period of fruit growth (organic acids, tannins) combine functions of protecting and deterring from fruit consumption and serve as precursors for secondary metabolism occurring later in fruit development (**Supplementary Figure S4**). In the middle stages of fruit development, ascorbic acid is accumulated, seed fatty acids are formed, color appears and softening begins, indicating the beginning of a switch from growth to ripening stage (**Supplementary Figure S4**). On ripening, major primary (e.g., sugars) and secondary metabolites are formed, coupled with further fruit softening and development of rich dark color due to anthocyanin biosynthesis, all in order to aid fruit attractiveness and seed dispersal (**Supplementary Figure S4**). Overall, the data indicate the magnitude of the changes in fruit phytochemistry throughout blackcurrant fruit development. As such they indicate the utility of ripening fruit as a model for the association of gene expression with the accumulation of specific fruit phytochemicals providing opportunities for the functional characterization of gene products. Transcriptome analysis of developing blackcurrant fruit has provided insight into processes linked to signaling and regulation in fruit ripening. Major patterns of gene expression have been identified and suggest the presence of diverse processes taking part throughout different stages of fruit development. Gene ontology analysis has emphasized this observation by providing an overview of divergent significant terms underpinning the diverse patterns of expression of significantly changing transcripts. These, similar to previously indicated patterns in phenotypic analysis of developing fruit, could be generalized into three main periods of fruit development; early fruit growth (stages 1–2), middle stages linked to further development processes (stages 3–4), and third ripening stage, linked to secondary metabolism (stages 5–6). Early fruit development was dominated by enrichment of GO terms with functions in cell processes (**Figure 8**) such as cell cycle, regulation of cell cycle and cell wall modification. This was consistent with active growth at the early stages of fruit development and may represent a key event in fleshy fruits associated with expansion processes and endoreduplication (McAtee et al., 2013). Fruit growth requires the combined presence of several growth-promoting plant hormones, such as auxin, gibberellins, cytokinins, and brassinosteroids (Ozga and Reinecke, 2003; Srivastava and Handa, 2005). This was reflected in another set of GO terms enriched at early fruit development stages. These included a range of auxin signaling and response proteins present under red light signal transduction and auxin signaling terms and was also significant in hormone targeted analysis by the presence of a range of IAA-related transcription factors (IAA7, IAA9, IAA16, IAA19, and IAA29) and PIN transporters maintaining auxin gradients, the presence of which is essential for growth in tomato (Pattison and Catala, 2012). Auxin, together with gibberellins, plays an important role during the growth phase by influencing cell enlargement (Csukasi et al., 2011), and indeed in blackcurrant a DELLA family protein RGL1 was present which is known in mango to function in the degradation of cell wall components affecting firmness (Dautt-Castro et al., 2015). Other hormone homologs expressed early in fruit development included abscisic acid NCED4, widely studied in climacteric and non-climacteric fruits as a protein altering fruit texture that may be involved in the initiation of ABA biosynthesis at the onset of fruit ripening (Mou et al., 2016). Abscisic acid has been associated with the expansion phase in tomato (Gillaspy et al., 1993) and ABA-deficient mutants have reduced fruit size (Nitsch et al., 2012). ABA has been widely acknowledged to regulate ethylene biosynthesis and signaling during fruit ripening, but the molecular mechanism underlying the interaction between these two hormones are largely unexplored. One ethylene response factor receptor (ERF 105) was highly expressed in the early stages of fruit development, but most of the identified homologs related to this hormone were observed at the ripening phase (**Figure 8**). Cytokinin regulators (CKI1, ARR9) and oxidases (CKX), in line with tomato (Kumar et al., 2014) and grape transcription patterns, had high expression in unripe fruit, which along with auxin, gibberellin, and abscisic acid emphasizes cytokinin involvement in berry set and growth (Fortes et al., 2015). High transcriptional activity was observed in the first stages of fruit development, and included transcription factors controlling developmental functions such as cell proliferation and differentiation (SCHIZORIZA, FRUITFULL), mitosis, cuticle development (SHINE, MYB16, HD-ZIP) fruit set and development (AGL7, AGL8), and seed development (**Figure 8**). As well as regulating developmental processes at early stages of ripening, some of those transcription factors may have important downstream regulatory functions in ripening, such as FRUITFULL which has functionality in the regulation of ethylene-independent aspects of tomato and bilberry fruit ripening (Jaakola et al., 2010; Fujisawa et al., 2014). Interestingly, HD-ZIP family homolog protein (ANL2, HDG1) expression coincided in blackcurrant with ethylene ACC Oxidase (ACO1). In tomato, HD-Zip HB-1 was shown to interact directly, and is necessary for ACO1 expression, and repression of it led to decrease in ethylene synthesis and delayed ripening (Lin et al., 2008). In tomato, however, ACO1 is expressed only in late stages of fruit ripening which might be one of the differences between ethylene regulation in climacteric and non-climacteric fruit. Additionally, patterns of expression of HD-Zip homologs in blackcurrant was similar to non-climacteric grape (Deluc et al., 2007).

Gene Ontology terms of transcripts highly expressed in the middle stages of fruit development (stages 3 and 4) indicated strong representation of the terms associated with post-embryonic development and development of reproductive structures (seed). The importance of seed development processes in middle stages was also underpinned by the presence of transcription factors encoding proteins homologous to those with functions in seed and embryo development such as ZF-HD – MATERNAL EMBRYO ARREST, WRKY2 – where mutants manifest distorted embryo growth (Ueda et al., 2011) and FUS3, a key regulator essential for embryo development in Arabidopsis (Wang and Perry, 2013) (**Figure 8**). This may indicate that, similar to Arabidopsis, complex cross talk and interactions among embryo transcription factors and their target processes exist, and additionally in blackcurrant those processes are significant for the middle stages of fruit development.

In the final stages of fruit development, similar to those of small green and large green fruit, GO terms associated in response to stimuli and particularly hormone regulation such as auxin (GO:0009733) prevailed. Also, homologs associated with jasmonate (at1g55020) and ethylene (ACC synthase) were upregulated. In hormone-targeted analysis abundance of ethylene related homologs was indeed recognized. This was seen by the presence of several ethylene receptor homologs (ETR2, EIN4, ERS1, **Figure 8**). This is similar to grape and pepper, and may provide evidence that the ethylene-mediated signaling pathway is active during ripening of those non-climacteric fruits (Lee et al., 2010; Fortes et al., 2015), although as non-climacteric fruit ethylene accumulation is not significant and abscisic acid is believed to have a stronger role during ripening (McAtee et al., 2013). It has been shown in tomato that ABA promotes ripening by inducing ethylene biosynthesis through upregulation of ethylene biosynthesis genes (Sun et al., 2012). Also in fruit that have lower requirements of ethylene to ripen, ABA homologs were highly represented in early and ripening stages of blackcurrant fruit and also in late stages (5–6), and included ABA synthesis AAO3 and response factors ABI1, ABF2, and AREB3 (**Figure 8**). The importance of ABA in fruit maturation is studied, for example, in some traits linked to the process such as color change. It has been indicated that in grape and strawberry, color change is strongly regulated by abscisic acid (Deytieux et al., 2004; Jia et al., 2011). This, consistent with phenotypic analysis of developing fruit, can be linked to secondary metabolism regulation in the final stages of fruit development as manifested in the current transcriptome analysis through an over-representation of GO terms linked to secondary metabolic processes (GO:0019748), aromatic compounds (GO:0019438), amino acid derivatives (GO:0042398), and phenylpropanoid metabolism (GO:0009698), with several homologs of glutathione *S*-transferases with roles in flavonoid transport (Petrussa et al., 2013; Zhao, 2015). The importance of secondary metabolism regulation in the final stages of fruit development was also confirmed by the presence of transcription factors such as WRKY23 that regulates biosynthesis of quercetin through auxin distribution (Grunewald et al., 2013). Several transcription factors were also related to stress response, including hypoxia (WRKY75), and it was shown that berry maturation was accompanied by enhanced stress-related metabolism, as in grape, which could be associated with specific anthocyanin accumulation (Degu et al., 2014). Interestingly, one of the jasmonate homologs MYC2, present in late stages of fruit development, was shown to have a key role in activating transcription factors such as WRKYs, MYBs, and ERFs, combining several processes already mentioned in late stages of fruit development such as ethylene signaling, secondary metabolism and color development.

Blackcurrant hormonal and regulatory networks have not been studied in detail previously and this study provides a first global outlook into *Ribes* networks controlling fruit ripening. Though still limited, understanding of the genetic and molecular complexes of fruit development is an essential foundation required for the development of fruit with enhanced quality. Knowledge presented here further expands genomic resources of *R. nigrum* and provides an opportunity for the future identification of key genes associated with the accumulation of specific quality components. In particular, the work lays the basis for gene discovery in complex biochemical pathways comprising enzymes encoded by multigene families. A key example is the pathway of anthocyanin biosynthesis that utilizes 2-oxoglutarate dependent dioxygenases, cytochrome P450s, glycosyltransferases and acyltransferases all of which are represented by large gene families.

## Author Contributions

DJ collected the samples, conducted the experimental work, analyzed the data, and contributed to the writing the manuscript. JM processed microarrays. DC designed qRT-PCR experiments. SG collected samples. SV conducted statistical analysis of microarray and metabolomics datasets. LM assembled genomic information and designed microarrays. PH analyzed array data. JA analyzed and annotated secondary metabolite data. RB and RH designed the study and drafted the manuscript.

## Conflict of Interest Statement

Lucozade Ribena Suntory (LRS) support a blackcurrant breeding programme at the James Hutton Institute for the development of cultivars to be used in the production of the blackcurrant cordial Ribena^TM^. LRS provided partial financial support for the Ph.D. studentship awarded to DJ. The work presented in this manuscript was undertaken at the James Hutton Institute (JHI) and was conceived and executed solely by JHI and James Hutton Limited staff. The manuscript was written without editorial comment from LRS. The remaining authors declare that the research was conducted in the absence of any commercial or financial relationships that could be construed as a potential conflict of interest.

## Footnotes

1 http://www.ebi.ac.uk/arrayexpress/

2 http://trinityrnaseq.sourceforge.net/

3 http://plantgdb.org/XGDB/phplib/download.php?GDB=Vv

4 https://earray.chem.agilent.com/earray/

5 http://www.ebi.ac.uk/arrayexpress/

6 http://bioinfo.cau.edu.cn/agriGO/analysis.php

## Supplementary Material

FIGURE S1 Dry matter content of developing blackcurrant fruit. Dry matter content was estimated following lyophilisation of a single sample comprising 100 fresh fruit as dry weight × 100/fresh weight.

FIGURE S2 Relative proportion of differentially abundant transcripts associated with specific GO terms at different stages of fruit development. White bars indicate the proportion of significantly differentially abundant transcripts within each GO term identifier amongst all of the transcripts differentially abundant at different stages of fruit development: small green **(A)**, large green **(B)**, green–red **(C)**, red–green **(D)**, red **(E)**, and ripe **(F)**. Black bars represent the proportion of genes within each GO-term identifier in the Arabidopsis genome relative to all identified genes in the Arabidopsis genome. GO term descriptions are provided in **Supplementary Table S4**.

FIGURE S3 Comparison of relative abundance of transcripts as estimated by microarray analysis and qRT-PCR. Transcripts were chosen as representative of each of the *K*-means patterns of expression as indicated in **Figure 5**. Relative abundance was estimated from the average normalized fluorescence of the appropriate microarray or by the 2^-ΔΔ*C*_t_^ method (Livak and Schmittgen, 2001) following qRT-PCR as described. Panels indicate the relative abundance of transcripts encoding lactate/malate dehydrogenase (comp13106, **A**), glycosyl hydrolase family 32 protein (comp14797, **B**), CYP450 family protein (comp1568, **C**), α-galactosidase 2 (comp17491, **D**), UDP-glucose dehydrogenase family protein (comp 21742, **E**) and sucrose synthase 3 (comp 1166, **F**). Error bars indicate the standard error estimated from three biological replicates.

FIGURE S4 Overview of changes in fruit chemistry during development. An illustration of the six stages of fruit development are shown at the top of the figure. Horizontal bars indicate the relative abundance of different groups of compounds during fruit development.

TABLE S1 Primers used for qRT-PCR.

TABLE S2 Change in relative abundance of chemicals at six different stages of fruit development.

TABLE S3 Transcripts exhibiting significant changes in abundance during fruit development.

TABLE S4 Significantly enriched gene ontology terms amongst transcripts assigned to six *K* means clusters dependent on changes in abundance during fruit development.

TABLE S5 Transcripts encoding transcription factors exhibiting significant differences in abundance during fruit development.

TABLE S6 Transcripts encoding proteins associated with hormone metabolism and signaling exhibiting significant differences in abundance during fruit development. Transcripts associated with metabolisms and signaling of cytokinin **(A)**, auxin **(B)**, gibberellin **(C)**, ethylene **(D)**, abscisic acid **(E)**, brassinosteroid **(F)**, jasmonate **(G)**, and salicylate **(H)** are listed.
