# Supplementary figures and images for: A Transcript and Metabolite Atlas of Blackcurrant Fruit Development Highlights Hormonal Regulation and Reveals the Role of Key Transcription Factors
Source: Front Plant Sci. 2018 Aug 24;9:1235. doi: 10.3389/fpls.2018.01235 (PMC6119775; doi:10.3389/fpls.2018.01235)

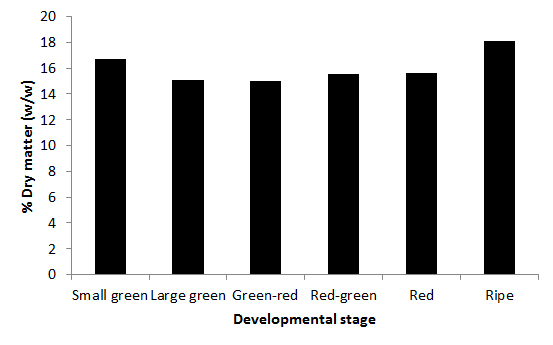

Supplement: FIGURE S1 — Dry matter content of developing blackcurrant fruit. Dry matter content was estimated following lyophilisation of a single sample comprising 100 fresh fruit as dry weight × 100/fresh weight. [file Image_1.JPEG]

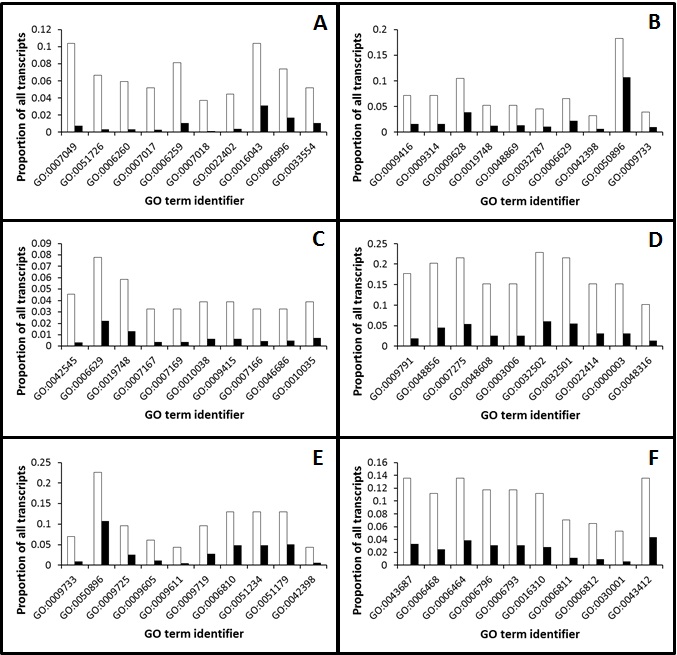

Supplement: FIGURE S2 — Relative proportion of differentially abundant transcripts associated with specific GO terms at different stages of fruit development. White bars indicate the proportion of significantly differentially abundant transcripts within each GO term identifier amongst all of the transcripts differentially abundant at different stages of fruit development: small green (A), large green (B), green–red (C), red–green (D), red (E), and ripe (F). Black bars represent the proportion of genes within each GO-term identifier in the Arabidopsis genome relative to all identified genes in the Arabidopsis genome. GO term descriptions are provided in Supplementary Table S4. [file Image_2.JPEG]

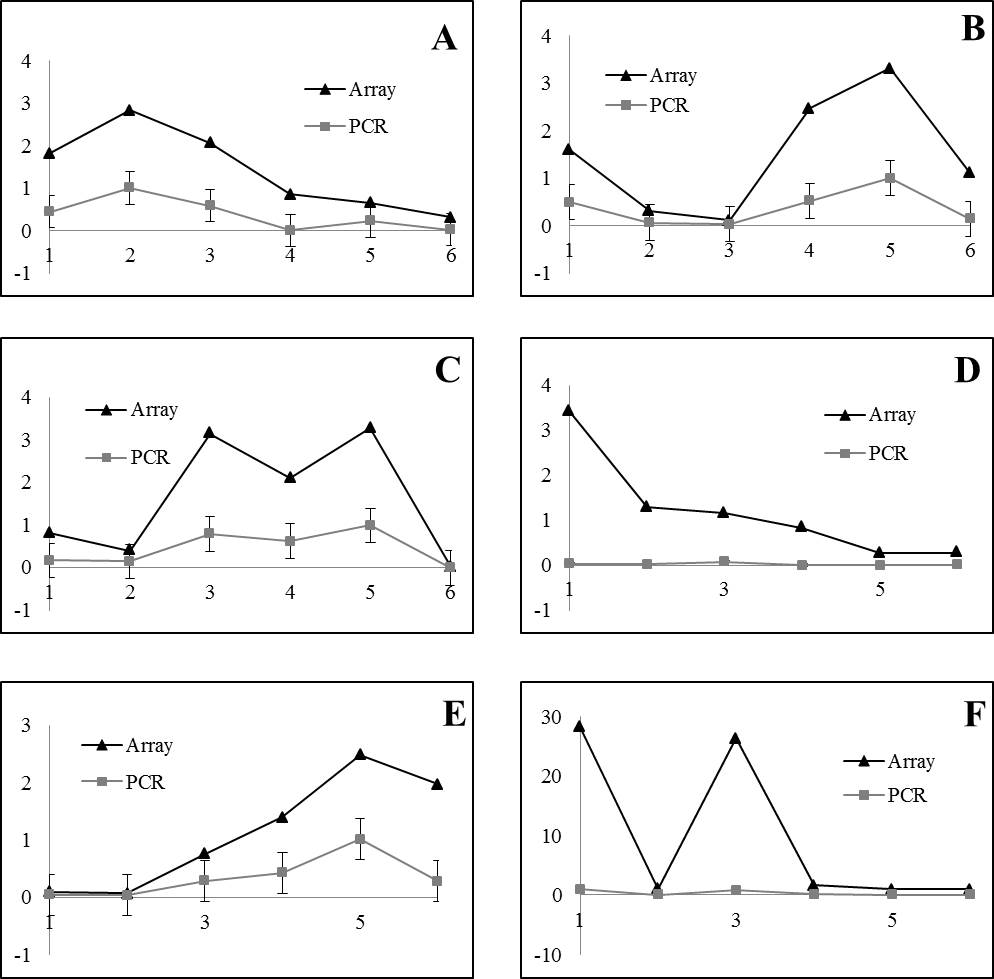

Supplement: FIGURE S3 — Comparison of relative abundance of transcripts as estimated by microarray analysis and qRT-PCR. Transcripts were chosen as representative of each of the K-means patterns of expression as indicated in Figure 5. Relative abundance was estimated from the average normalized fluorescence of the appropriate microarray or by the 2-ΔΔCt method (Livak and Schmittgen, 2001) following qRT-PCR as described. Panels indicate the relative abundance of transcripts encoding lactate/malate dehydrogenase (comp13106, A), glycosyl hydrolase family 32 protein (comp14797, B), CYP450 family protein (comp1568, C), α-galactosidase 2 (comp17491, D), UDP-glucose dehydrogenase family protein (comp 21742, E) and sucrose synthase 3 (comp 1166, F). Error bars indicate the standard error estimated from three biological replicates. [file Image_3.JPEG]

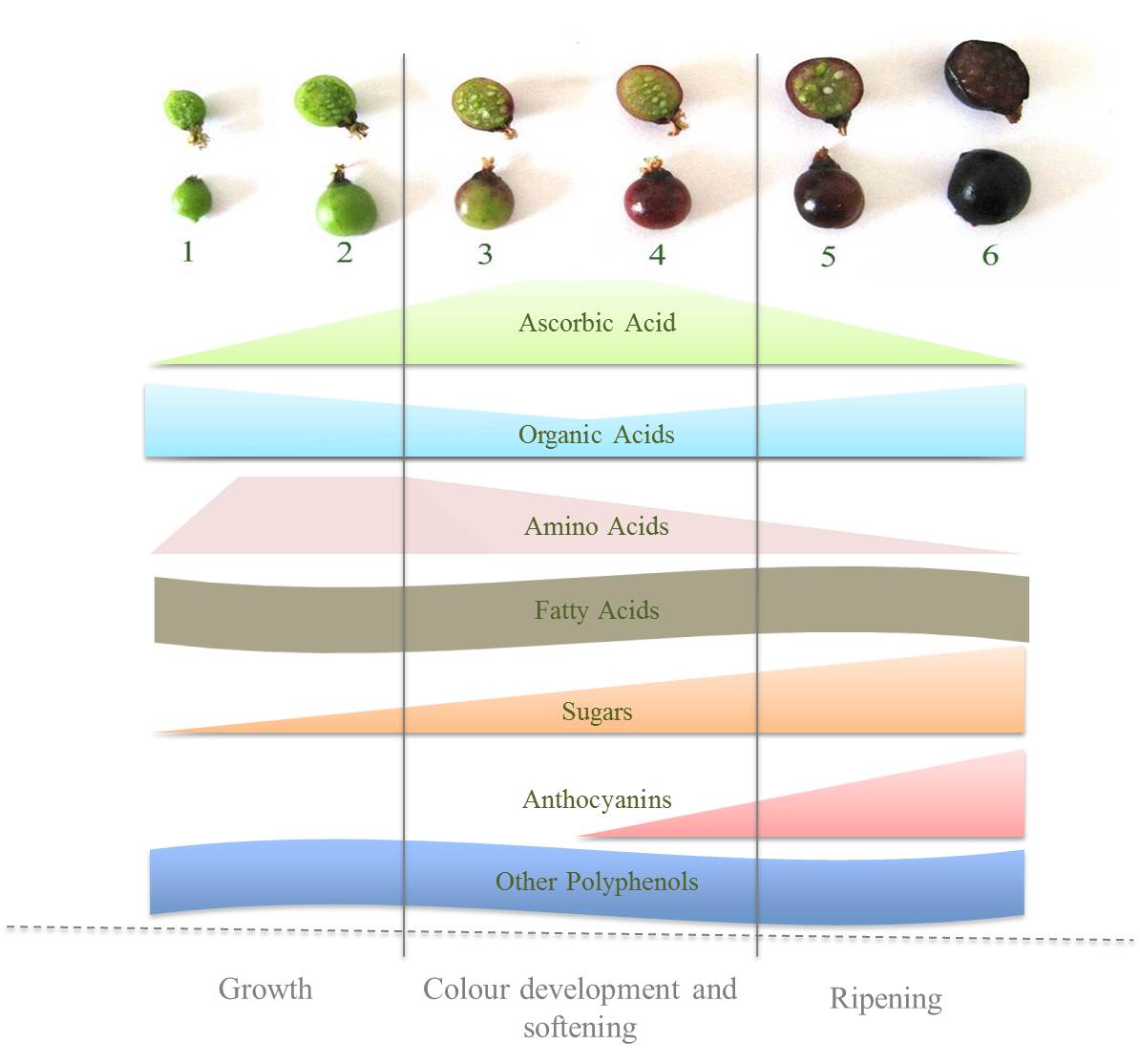

Supplement: FIGURE S4 — Overview of changes in fruit chemistry during development. An illustration of the six stages of fruit development are shown at the top of the figure. Horizontal bars indicate the relative abundance of different groups of compounds during fruit development. [file Image_4.JPEG]
